# *Artemisia absinthium* L. ethanol extract inhibits the growth of gastrointestinal cancer cells by inducing apoptosis and mitochondria-dependent pathway

**DOI:** 10.3389/fonc.2025.1644498

**Published:** 2025-10-27

**Authors:** Ting Li, Shaoqi Yu, Zihang Ma, Jinyao Li, Lijie Xia, Yan Feng

**Affiliations:** ^1^ Department of Gastroenterology, People’s Hospital of Xinjiang Uygur Autonomous Region, Urumqi, China; ^2^ Xinjiang Clinical Research Center for Digestive Disease, Urumqi, China; ^3^ Xinjiang Key Laboratory of Biological Resources and Genetic Engineering, College of Life Science and Technology, Xinjiang University, Urumqi, China

**Keywords:** gastrointestinal cancer, *Artemisia absinthium* L., anti-tumor activity, luteolinidin, apoptosis

## Abstract

**Objectives:**

*Artemisia absinthium* L. has a long history in the treatment of gastrointestinal cancer (GIC), but its molecular mechanisms remain unclear.

**Materials and methods:**

We identified and validated the active components and key targets in *A. absinthium* for the treatment of GIC by LC-MS and Network analysis. The antitumor effect of *A. absinthium* ethanol extract (AAEM-V) against gastrointestinal tract cancer *in vitro* and *in vivo* as well as the anticancer activity of the active ingredient, Luteolinidin, were further evaluated.

**Results:**

AAEM-V exhibited good anticancer activity *in vitro* and *in vivo*. The active ingredient Luteolinidin was taken to intersect with the targets of gastric and colorectal cancers, and 69 common targets were identified. A total of three core targets, SRC, EGFR, and AKT1, were screened according to PPI and molecularly docked with Luteolinidin, and it was found that their binding played a key role in the treatment of tumors. Meanwhile, its active ingredient Luteolinidin was found to induce ROS proliferation to promote apoptosis and prevent cells from entering S phase.

**Conclusion:**

These findings not only explored the anti-gastrointestinal cancer chemical properties of *A. absinthium*, but also provided a new research direction for the active ingredients and mechanism of action of traditional Chinese medicine.

## 1 Introduction

Gastrointestinal cancer (GIC) is a significant worldwide health issue, encompassing malignant neoplasms such as esophageal, gastric, and colorectal cancers, with high morbidity and mortality rates, constituting a substantial threat to public health (1, 2). Traditional therapies, including surgery, chemotherapy, radiation and targeted therapies, remain the mainstay of treatment of gastrointestinal cancers (3, 4). Nevertheless, the deleterious side effects and unfavorable prognosis associated with the postoperative period can be a source of great suffering for many patients (5). The growth of GIC is affected by various elements, such as genetic makeup, environmental factors, lifestyle decisions, and the microbiome’s structure (6–8). Primary GICs have a slow onset and cannot be detected, but develop rapidly and have a high degree of malignancy (9, 10). At the time of clinical diagnosis, many patients with advanced GI cancer would have brain metastases (11, 12). Gastrointestinal cancers include esophageal, stomach and colorectal cancers (13). Gastric carcinoma, an issue of paramount significance to global public health, stands as a predominant contributor to cancer - related mortality on a global scale (14, 15). The pathogenesis of this malignancy is intricately intertwined with a multiplicity of factors, encompassing dietary practices, Helicobacter pylori infection, the presence of precancerous lesions, and both hereditary and genetic predispositions (16, 17). H. pylori infection has been unequivocally established as a significant and well-documented risk factor for the development of gastric cancer, with a global prevalence of up to 90%, and a direct correlation with the occurrence of distal gastric cancer (17, 18). Colorectal cancer is one of the most prevalent malignant tumors worldwide, and its pathogenesis is complex and varied (19, 20). The 2020 China Cancer Statistical Report shows that there were 555,000 new cases of colorectal cancer and 286,000 deaths in China (21). Moreover, the long-term utilization of chemotherapeutic agents such as fluorouracil, platinum, and paclitaxel is associated with the potential for severe toxic side effects and the development of drug resistance (22). Consequently, there is an urgent necessity to identify new drugs that exhibit both high safety and efficacy as novel therapeutic options.

As a unique biomedical resource, traditional Chinese medicine has been widely used in the prevention and treatment of GIC (23–25). Artemisia is a perennial, biennial or annual herb or shrub distributed in the temperate zones of Asia, Europe and North America, containing 200–400 species (26). It exhibits a broad spectrum of pharmacological activities, encompassing anti-ulcer, antineoplastic, hepatoprotective, and anti-diabetic effects (27). *A. absinthium* is a plant belonging to the genus Artemisia, commonly known as wormwood, which is produced in Xinjiang, China, South Asia, North America, etc. (28). The active ingredients present in *A. absinthium* have led to its extensive utilization in the treatment of various tumors; however, the target and mechanism of its anti-gastrointestinal cancer effects remain to be fully elucidated.

A plethora of medicinal materials and complex components are present in Traditional Chinese Medicine (TCM) compounds, which complicates the elucidation of their mechanisms of action. Liquid chromatography-mass spectrometry (LC-MS) tools are increasingly utilized in the drug discovery stage (29, 30). Network analysis offers a novel approach to the study of complex traditional Chinese medicine systems (31). Network analysis is an interdisciplinary field that integrates system biology, bioinformatics, multidirectional pharmacology and other related disciplines, thereby elucidating the intricate network of interactions between drugs, genes, targets and diseases (32). It is capable of systematically elucidating the mechanism of traditional Chinese medicine by demonstrating the relationship among drugs, targets and diseases, thereby generating new concepts for the advancement of traditional Chinese medicine and novel drugs (33). Molecular docking represents a theoretical simulation technique that principally investigates intermolecular interactions and predicts their binding mode and affinity (34, 35). It can be utilized to ascertain the binding site and binding affinity of drugs to target proteins (36).

In the present study, we firstly explored the anti-gastrointestinal tumor activity of AAEM-V at the cellular level and mouse model level, and secondly identified the compounds in the ethanolic extract of *A. absinthium* using LC-MS, and Luteolinidin, as a potentially active substance in AAEM-V, analyzed the potential molecular network mechanism of the active component using network biology, and verified the *in vitro* anti-gastrointestinal tumor activity of the compound. The results of this study will provide a new theoretical basis and direction for the systematic advancement of gastrointestinal tumor research.

## 2 Materials and methods

### 2.1 Preparation of AAEM-V

*A. absinthium* components purified by 85% ethanol with macroporous resin (AAEM-V) is obtained from *A. absinthium* ethanol extract (AAEE). Absorption experiments of AAEE on AB-8 resin were conducted by infusing 60 mg/mL of AAEE 1/10 BV water solution into the resin column. After absorption, resins were added with water and different concentrations of ethanol (0%, 30%, 50%, 70%, 85%, 100%). The 85% ethanol eluent phase was collected, condensed with a rotary evaporator, and then dissolved in dimethyl sulfoxide (DMSO) to a final concentration of 50 mg/mL and filtered through a 0.22 μm filter.

### 2.2 Metabolites extraction

50 mg of sample were applied to extraction procedure, and extracted with 800 μL of methanol included internal standard(2.8mg/mL, DL-o-Chlorophenylalanine). And all samples were grinded to fine powder using Grinding Mill at 65 Hz for 90 s. Then the samples were ultrasonicated for 30 min, by 40 KHz and let stand for 1 hour at -20°C. The samples were centrifuged at 12000 rpm and 4°C for 15 min.200 μL of supernatant was transferred to vial for LC-MS analysis.

### 2.3 LC/MS analysis

Analysis platform: LC-Q/TOF-MS (Agilent,1290 Infinity LC, 6530 UHD and Accurate-Mass Q-TOF/MS). Column: Waters ACQUITY UPLC@HSS T3, 2.5um 100*2.1mm. Chromatographic separation conditions: Column temperature: 40°C; Flow rate: 0.4 mL/min; Mobile phase A: water+0.1% formic acid; Mobile phase B: acetonitrile+0.1% formic acid; Injection volume: 4 μL; Automatic injector temperature: 4°C. The gradient elution procedure is shown in **Table 1**. The mass spectrometry analysis was performed in both positive and negative ion modes with optimized parameters: capillary voltages of 4 kV (positive) and 3.5 kV (negative), a scan range of *m/z* 100–1000, and dynamic collision energy for MS/MS fragmentation. The mass spectrometer was calibrated in real-time using lock mass ions: 121.0509 and 922.0098 (positive mode) and 119.0363 and 966.0007 (negative mode) to ensure mass accuracy below 1 ppm.

**Table 1:** The gradient of mobile phase.

Time (min)	Flow (mL/min)	Pressure limit (bar)	Solv ratio B (%)
0	0.4	95	5
2	0.4	95	5
13	0.4	5	95
16	0.4	5	95

### 2.4 Network analysis of active ingredients

TCMSP (https://tcmsp-e.com/), PubChem (https://pubchem.ncbi.nlm.nih.gov/) and SwissTargetPrediction (http://www.swisstargetprediction.ch/) databases were used to predict the target genes corresponding to the active ingredients in order to collect a comprehensive collection of compound targets. First, compounds were screened using TCMSP based on oral bioavailability (OB ≥ 30%) and drug-like properties (DL ≥ 0.18). Then, “gastric cancer” and “colorectal cancer” were entered into the GeneCards (https://www.genecards.org/) and OMIM databases (https://www.omim.org/), respectively, to search for and collect disease targets. Input the compound targets and disease targets into Venny 2.1.0 (https://bioinfogp.cnb.csic.es/) to draw a Wayne diagram and obtain the intersecting target genes. The intersecting targets were imported into STRING database (https://cn.string-db.org/), and the free targets were eliminated and imported into Cytoscape 3.9.1 software for analysis, and the key target genes of compounds acting on diseases were screened out according to the Degree value ranking and PPI networks were made. The potential core targets of drug treatment diseases were imported in DAVID database and analyzed by GO BP, GO CC, GO MF, KEGG enrichment, respectively, and the results were outputted as bar chart and bubble chart results through the mapping website of Bioinformatics (http://www.bioinformatics.com.cn/). Component-target-signaling pathway network diagrams were constructed on the basis of the included components, targets, and signaling pathways, and the organized data were imported into Cytoscape 3.9.1 software for network diagrams.

### 2.5 Key ingredient - core target molecular docking

To explore the interactions between targets and key compounds, molecular docking was performed. Target protein structures were retrieved from the RCSB PDB database (http://www.rcsb.org/), while 2D structures of AAEM-V active components were obtained from the PubChem database. The structural optimization of key com-pounds and targets—including 3D chemical structure generation, energy minimization, and format conversion—was achieved using ChemBio Draw 3D and Autodock Tool. For protein preparation, PyMol was employed to remove ligands, correct protein conformations, and eliminate water molecules. Docking procedures were conducted via Autodock Vina, with the molecule exhibiting the lowest binding energy in the docked conformation selected for analysis.

### 2.6 Cell culture

The human gastric cancer cell lines BGC823 and murine colorectal cancer cell lines CT26 were obtained from the Xinjiang Key Laboratory of Biological Resources and Genetic Engineering, Xinjiang University (Urumqi, Xinjiang, China). The cells were maintained in RPMI 1640 medium (Gibco, Thermo Fisher Scientific, Waltham, MA, USA) containing 10% heat-inactivated fetal calf serum, (MRC, Changzhou, China), 1% L-glutamine (100 mM), 100 U/mL penicillin and 100 μg/mL streptomycin. All cells were cultured at 37°C in a 5% CO_2_ incubator. All cell lines used in this study were regularly tested using a mycoplasma detection kit, and all experiments were conducted after the cells were confirmed to be mycoplasma-negative.

### 2.7 MTT bioassay

The assessment of cell viability was conducted through the utilization of the MTT method. The cells were inoculated into 96-well cell culture plates at a density of 5×103 cells/well, and 100 μL of cell culture medium was added. This was replaced with fresh medium containing different concentrations of drug and 0.2% DMSO after 24 h. Cisplatin (30 μg/mL) was used as a positive control. After 24 h incubation, the supernatant was removed by centrifugation. 100 μL MTT (0.5 mg/mL) was added to each well and incubated for 4 h at 37°C in the dark. The supernatants were removed by centrifugation, dissolved with 150 μL DMSO, and the OD value of each well was detected at 490 nm.

### 2.8 Apoptosis

The analysis of apoptosis was conducted by utilizing the Annexin V-FITC Apoptosis Detection Kit. The cells were treated with various concentrations of drug and 0.2% DMSO for a duration of 24 h. Subsequently, the cells were harvested and incubated with PI and FITC for a period of 15 minutes in a dark environment. The determination of apoptotic cells was con-ducted through the utilization of flow cytometry.

### 2.9 Hoechst 33258 staining

Cells were treated with drug, DMSO and cisplatin for 24 hours, washed with PBS, fixed with 4% paraformaldehyde and stained with Hoechst 33258 staining solution. The cells were observed by inverted fluorescence microscope.

### 2.10 ROS

Cells were treated with drug, DMSO and cisplatin for 24 h. Cells were washed with PBS and then stained with 10 µM of the fluorescent probe DCFH-DA for 20 min. After three washes with PBS, the fluorescence intensity of the cells was measured by flow cytometry.

### 2.11 Mitochondrial membrane potential (Δψm)

Cells were treated with drug, DMSO and cisplatin for 24 h. After two washes with PBS, cells were resuspended with 300 µL of JC-1 staining solution and incubated at 37°C for 30 min, followed by two washes with PBS before detection and analysis by flow cytometry.

### 2.12 Cell cycle

Cells were treated with drug, DMSO and cisplatin for 24 h. Cells were collected, washed with PBS and fixed in 70% ethanol. After rinsing twice with PBS, the cells were stained with PI at 37°C for 30 min, and then the samples were analyzed by flow cytometry.

### 2.13 *In vivo* experiment

BALB/c mice, male, weighing 20 ± 2 g, were purchased from the Experimental Animal Center of Xinjiang Medical University and were supervised by the Experimental Ethics Committee of Xinjiang University (approval number: XJUAE-2024-017). They were housed in the animal facility of the School of Life Sciences and Technology at Xinjiang University, where they had free access to food, and the housing conditions complied with all ethical standards. After a one-week acclimatization period, CT26 cells (at a dose of 1×10^6^ cells per mouse) were injected into BALB/c mice via subcutaneous injection. Subsequently, tumor-positive mice were randomly divided into six groups(n=7), each containing seven mice. The selection of the internal dose followed the body surface area (BSA) conversion principle. After a seven-day observation period, tumor-positive mice were treated with dimethyl sulfoxide (DMSO), AAEM-V (at doses of 10 mg/kg, 50 mg/kg, and 100 mg/kg), and the chemotherapy drug cisplatin (at a dose of 5 mg/kg). Tumor size was measured using a Vernier caliper, and tumor volume was calculated using the following formula: Tumor volume (mm³) = (length × width²)/2.

### 2.14 Statistical analysis

All data were analyzed statistically using GraphPad Prism 9.4.0. Statistical tests were performed using one-way analysis of variance (ANOVA). If the p-value of the test result was less than 0.05, the difference between groups was considered statistically significant. The test results are presented as “mean ± standard deviation,” with n=3.

## 3 Results

### 3.1 AAEM-V inhibits the growth of GIC *in vitro*

**Figure 1:**
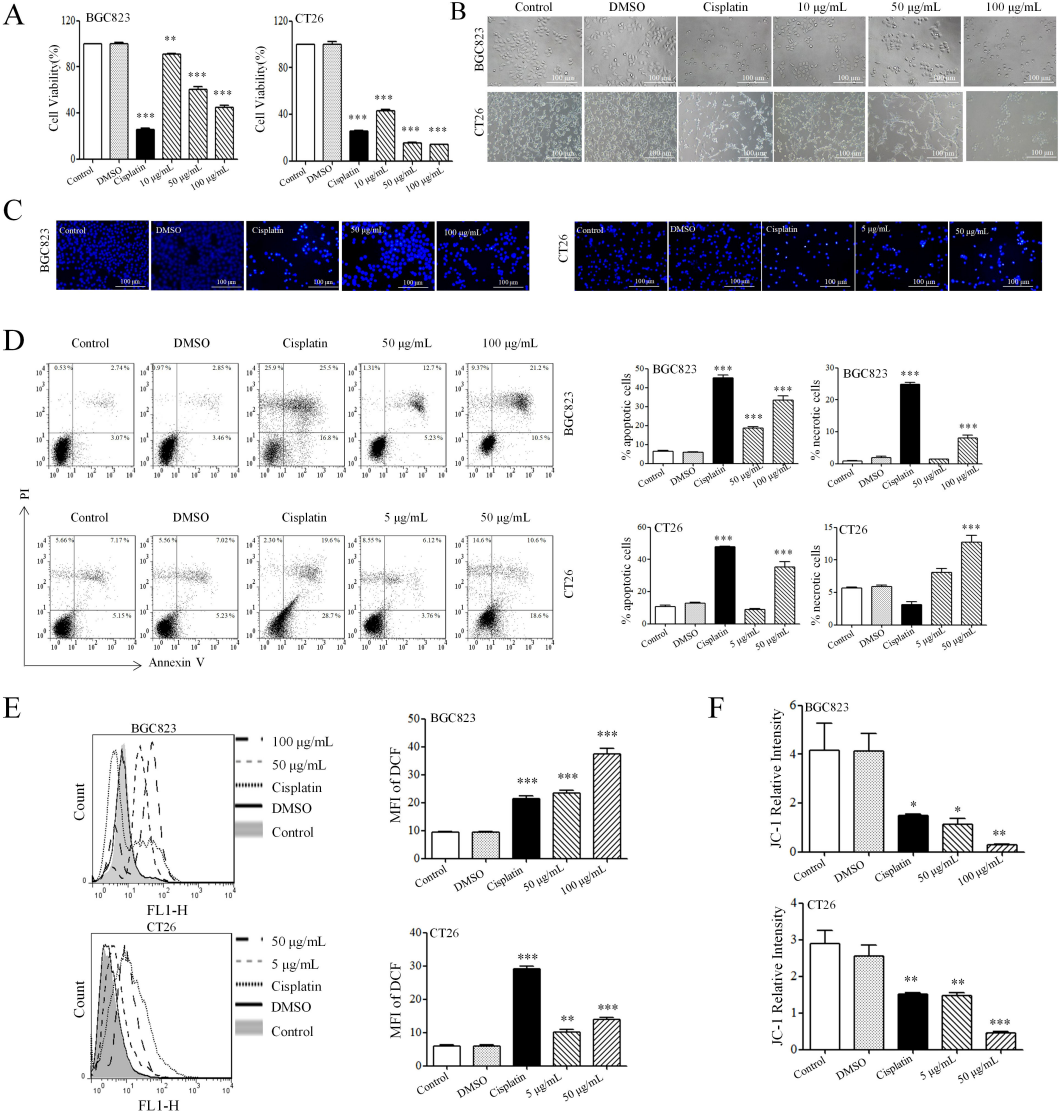
AAEM-V inhibits the growth of GIC *in vitro*. **(A)** The viability of cells was detected by MTT as-say. **(B)** The morphology of cells was observed by inverted microscopy. **(C)** The effect of Hoechst 33342 staining on cell chromatin was observed under an inverted fluorescence microscope. **(D)** After staining with Annexin V and PI, the cells were analyzed by flow cytometry. **(E)** Cellular ROS levels were detected by flow cytometry. **(F)** Statistical plot of mitochondrial membrane potential detection. Data were analyzed by one-way ANOVA, compared with control, *p<0.05, **p<0.01, ***p<0.001.

To evaluate the effect of AAEM-V on the growth of gastrointestinal tumor cells *in vitro*, BGC823 and CT26 cell lines were selected for MTT assay. The **Figure 1A** results showed that AAEM-V could significantly inhibit the proliferative ability of BGC823 and CT26 cells in a concentration-dependent manner, and 24 h semi-inhibitory concentration (IC_50_) values were 79.03 μg/mL and 6.794 μg/mL, respectively. As shown in **Figure 1B**, after 24 hours of AAEM-V treatment of the cells, the cell morphology changed significantly, and the cells were gradually wrinkled and rounded with the increase of drug concentration, and the number of cells decreased. Flow cytometry and inverted fluorescence microscopy were further utilized to assess the apoptotic effects of AAEM-V treatment in both quantitative and qualitative aspects in BGC823 and CT26 cell lines. Normal cell nuclei exhibit bright blue fluorescence, and the morphology and structure of the nuclei are clearly visible. In contrast, in the AAEM-V-treated group, as the drug concentration increases, the structure of the nuclei changes, the fluorescence becomes uneven, the chromatin aggregates and migrates toward the nuclear membrane, and some nuclei lyse into multiple dense fluorescent bodies (apoptotic bodies) (**Figure 1C**). Flow cytometry results showed that both low and high concentrations inhibited the proliferation of both cell lines in a dose-dependent manner compared with the control (**Figure 1D**).

ROS is a highly reactive molecule produced intracellularly that plays an important role in apoptosis. DCFH-DA (2’,7’-Dichlorodihydrofluorescein diacetate) was utilized as a fluorescent probe, DCFH-DA itself is non-fluorescent but can freely cross the cell membrane, and intra-cellular ROS oxidize DCFH to generate fluorescent DCF. The results of staining showed that AAEM-V-treated cells showed fluorescence enhancement in a dose-dependent manner (**Figure 1E**). When too much ROS are generated, it leads to a decrease in mitochondrial membrane potential and an increase in the permeability of the inner mitochondrial membrane, releasing proteins such as cytochrome C in the mitochondria, which in turn activates caspase proteins and triggers apoptosis. As shown in **Figure 1F**, the relative fluorescence intensity of JC-1 in cells treated with AAEM-V was reduced, indicating that the drug could significantly induce a decrease in mitochondrial membrane potential.

### 3.2 AAEM-V inhibits colorectal cancer development *in vivo*

**Figure 2:**
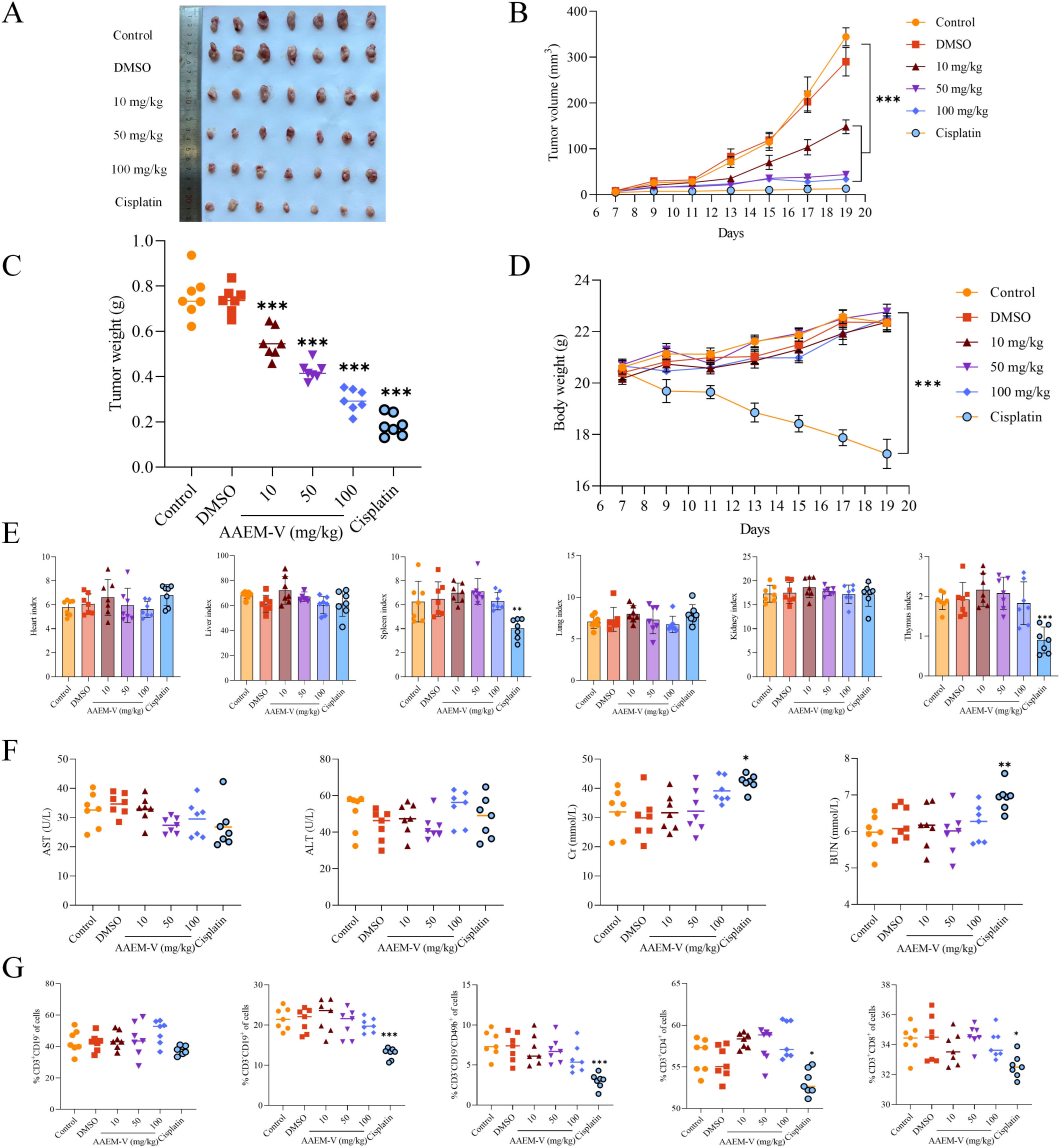
AAEM-V inhibits colorectal cancer development *in vivo*. **(A)** Anatomy of a mouse tumor. **(B)** Tumor volume changes in mice. **(C)** Tumor weight changes. **(D)** Mouse body weight changes. **(E)** Mouse organ index. **(F)** Liver and kidney function tests. **(G)** Detection of immune cell indices in the spleen of mice. Data were analyzed by one-way ANOVA, compared with control, *p<0.05, **p<0.01, ***p<0.001.

To further evaluate the anti-tumor activity of AAEM-V *in vivo*, we injected low, medium, and high (10 mg/kg, 50 mg/kg, 100 mg/kg) doses of AAEM-V every 2 days for a total of 14 days using the CT26 hormonal mouse model, while cisplatin (5 mg/kg) was used as a positive control. Compared with the control, the AAEM-V-treated group and the cisplatin-treated group were able to significantly (p<0.001) inhibit tumor growth in mice *in vivo* with a positive correlation with con-centration (**Figures 2A–C**). However, the body weight of mice in the cisplatin group was significantly (p<0.001) reduced compared to the control, on the contrary, AAEM-V treatment had no effect on the body weight of mice (**Figure 2D**), suggesting that cis-platin induced severe systemic toxicity, whereas AAEM-V had no significant toxic effects on the growth of mice. Meanwhile, there was no significant change in the organ index of mice after AAEM-V treatment, but the thymus and spleen in the cisplatin group were significantly reduced compared with control, further indicating that AAEM-V had no significant toxic effects on mice, and that Ran cisplatin was highly toxic (**Figure 2E**). Liver and renal function tests are important for assessing liver and kidney health. The test results showed that there was no significant difference in the serum levels of ALT, AST, BUN, and Cr in the AAEM-V-treated group of mice com-pared with the control (**Figure 2F**). Immune cells represent the core com-ponent of the immune system, performing multiple key functions such as defense against pathogens, maintenance of internal environmental stability, and surveillance of abnormal cells. Further tests on the splenic immune indexes of mice revealed that AAEM-V had no significant effect on the immune cells of mice (**Figure 2G**). In contrast, cisplatin reduced the proportions of B cells and NK cells, while simultaneously decreasing the numbers of CD4^+^ T cells and CD8^+^ T cells. The findings of this study indicate that AAEM-V exhibits lower toxicity and higher safety compared with the chemotherapeutic agent cisplatin, thereby suggesting its potential as a therapeutic strategy for colorectal cancer treatment.

### 3.3 Component identification of *A. absinthium*

**Figure 3:**
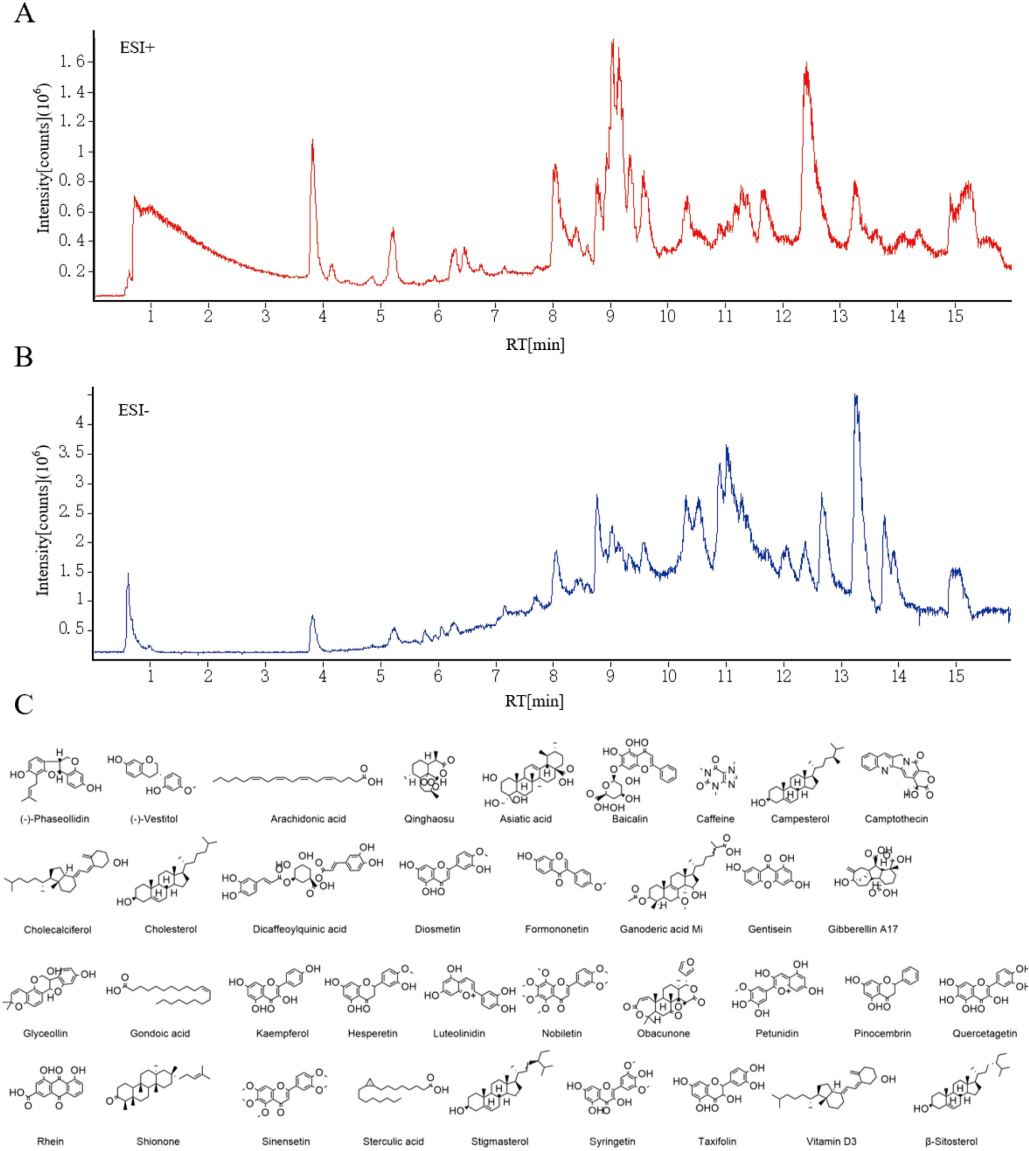
**(A)** Positive ion mode. **(B)** Negative ion mode. **(C)** The structure information of 36 active compounds from A. absinthium.

The Compound compositions of *A. absinthium* were explored by the LC-MS both in positive and negative modes, as shown in **Figures 3A, B**. The 444 compounds were identified by LC-MS, including amino acids, flavonoids, terpenoids, organic acids and so on. Combined with the Traditional Chinese Medicine Systematic Pharmacology Database and Analysis Platform (TCMSP), 36 compounds were selected (**Figure 3C**). These compounds may be key candidates for further exploration of their pharmacological activities and potential therapeutic applications.

### 3.4 Shared targets of drugs and diseases

**Figure 4:**
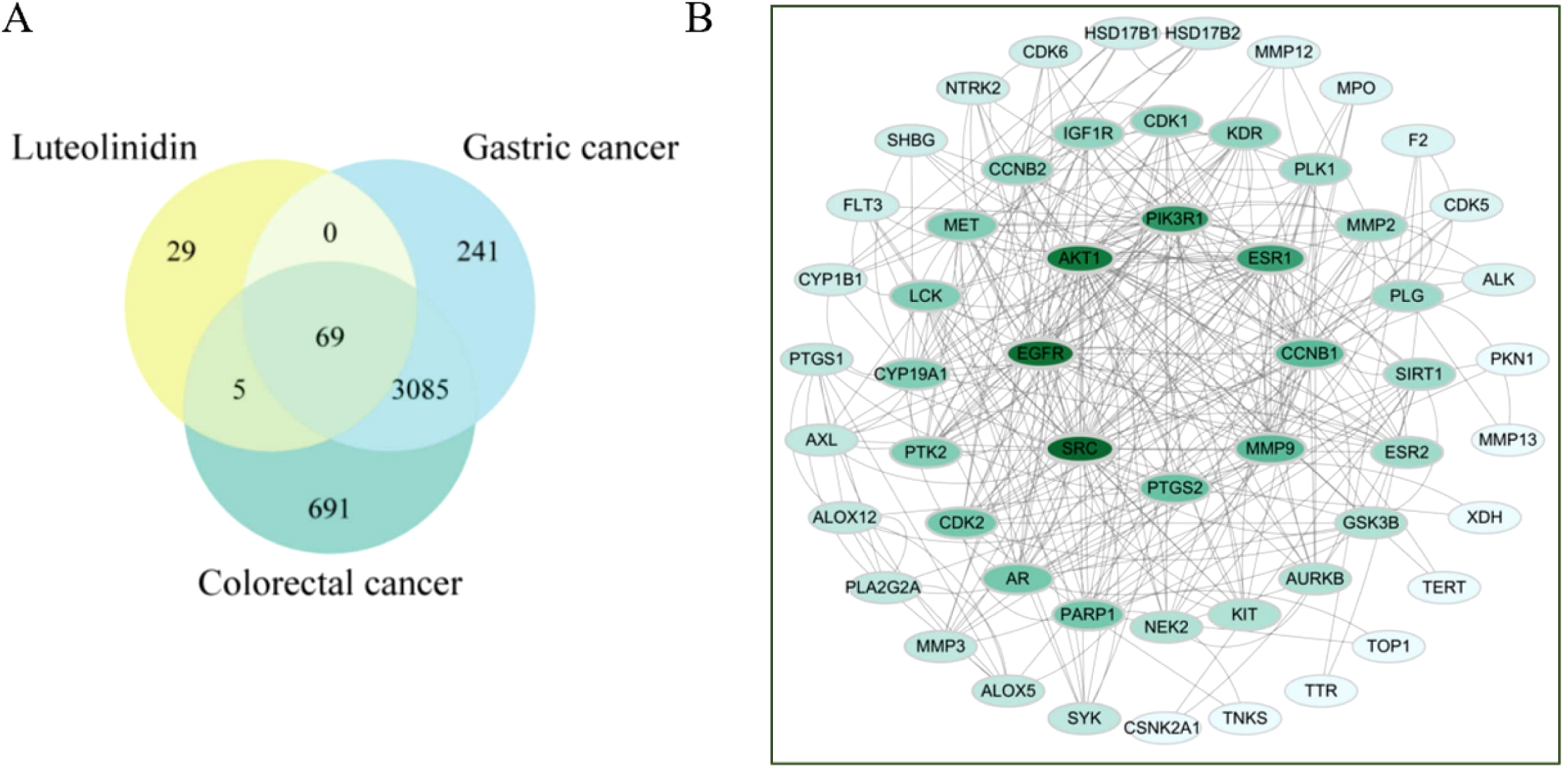
**(A)** Venn diagram of the active compounds and gastrointestinal cancer. **(B)** Shared Target Inter-active PPI Network.

From the 36 compounds screened, one key bioactive compound, Luteolinidin (yellow circle), was identified to interact with gastric (blue circle) and colorectal cancer (green circle) -related targets, yielding a total of 69 putative targets (**Figure 4A**). Using the STRING database for protein-protein interaction (PPI) network analysis, the resultant PPI network comprised 69 nodes and 164 edges, with an average node degree of 4.75 (**Figure 4B**). Topological analysis of the PPI network revealed that SRC, EGFR, AKT1, PIK3R1, ESR1, CCNB1, MMP9, and PTGS2 emerged as hub proteins, suggesting their central roles in gastrointestinal tumorigenesis.

### 3.5 Enrichment analysis of GO and KEGG pathway

**Figure 5:**
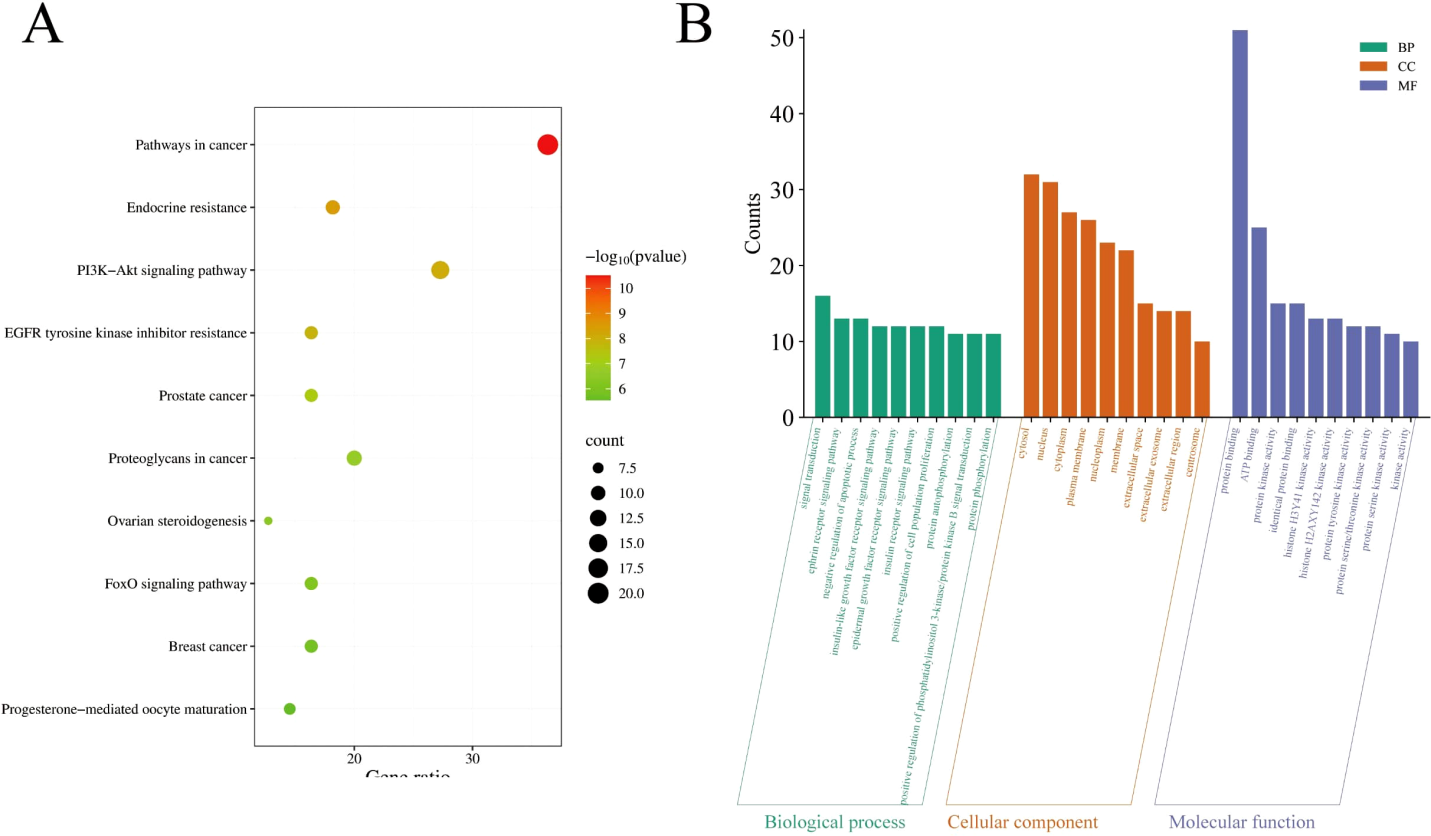
**(A)** The top 20 pathways for Kyoto Encyclopedia of Genes and Genomes enrichment analysis. **(B)** Gene Ontology enrichment analysis.

KEGG (Kyoto Encyclopedia of Genes and Genomes) and GO (Gene Ontology) enrichment analyses are core bioinformatics tools for revealing gene functions and signaling pathway mechanisms. KEGG pathway enrichment and GO enrichment analyses of the core targets were performed using the DAVID platform. A total of 87 pathways directly related to cancer development and progression were identified (P < 0.05). The top 10 pathways ranked by P-value are shown in **Figure 5A**, which were Pathways in cancer, Endocrine resistance, PI3K-Akt signaling pathway, EGFR tyrosine kinase inhibitor resistance, Prostate cancer, Proteoglycans in cancer, Ovarian steroidogenesis, FoxO signaling pathway, Breast cancer, and Progesterone-mediated oocyte maturation. The top 10 highly enriched GO categories in biological processes (BPs), molecular functions (MFs), and cellular components (CCs) are presented in **Figure 5B**. The results showed that the top three most enriched BP categories were signal transduction, ephrin receptor signaling pathway, and negative regulation of apoptotic process. In the CC category, the top three enrichments were cytosol, nucleus, and cytoplasm. In the MF category, the top three enrichments were protein binding, ATP binding, and protein kinase activity. Based on the enrichment analysis results, *A. absinthium* was suggested to target multiple functional and biological factors in GIC, although its specific role requires validation through further mechanistic studies.

### 3.6 Construction of active ingredient-shared key target- signal pathway network map

**Figure 6:**
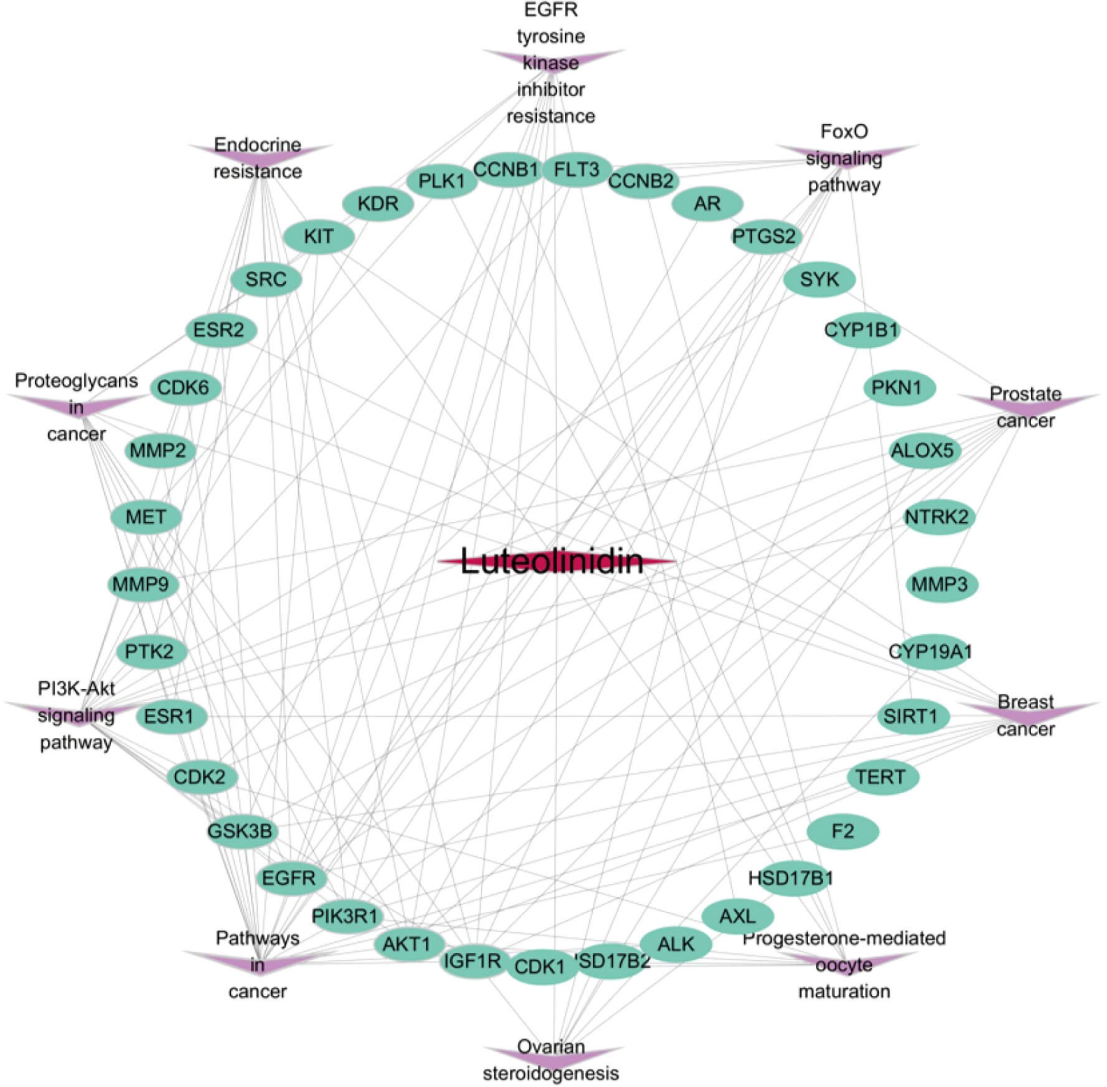
Component (Luteolinidin)-target-pathway network diagram.

To systematically dissect the molecular mechanisms of Luteolinidin against GI tumors, we constructed a compound-target-pathway network using Cytoscape 3.7.2 (**Figure 6**). In this network, the rose-red diamond denotes the bioactive compound (Luteolinidin), green ellipses represent core targets, and purple V-shaped nodes signify target-enriched pathways. Network topology analysis revealed that Luteolinidin exhibits polypharmacological properties by interacting with multiple targets, each of which participates in diverse signaling cascades. These findings suggest a potential synergistic effect of Luteolinidin across different biological processes, providing a molecular basis for its therapeutic efficacy against GI malignancies.

### 3.7 Molecular docking of key active ingredients to core targets

**Figure 7:**
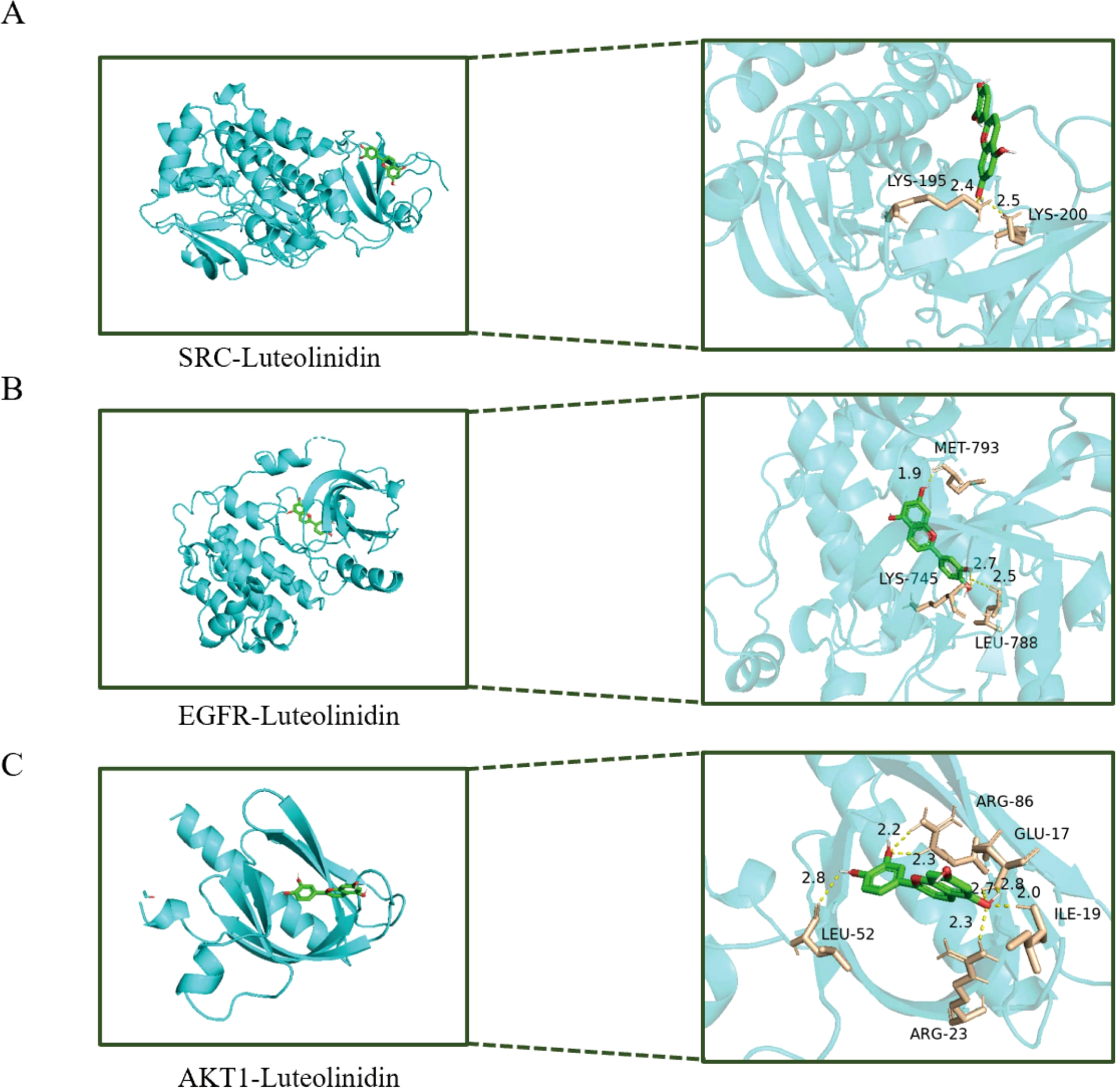
Molecular docking diagram of bioactive compounds to core targets. **(A)** SRC-Luteolinidin. **(B)** EGFR-Luteolinidin. **(C)** AKT1-Luteolinidin.

PPI plays critical roles in various biological processes and are emerging as promising therapeutic targets. Through PPI network analysis, we ranked the genes based on their degree values. SRC, EGFR, and AKT1 are all at the top of the list, indicating that they play a crucial hub role in the network. Secondly, we reviewed the results of the KEGG pathway enrichment analysis. Among the significantly enriched pathways, the ‘PI3K-Akt signaling pathway’ is one of the most prominent and significant cancer-related pathways, with AKT1 serving as a central node in this pathway. EGFR is a key driver of the ‘EGFR tyrosine kinase inhibitor resistance’ pathway and upstream RTK signaling. SRC is a well-known oncogene that plays a central role in various cancer signaling pathways. These three targets directly point to the most significantly enriched pathways we identified, thus warranting higher validation priority. In this study, we performed molecular docking simulations (**Figure 7**) to investigate the binding modes and affinities between key core targets (SRC, EGFR, AKT1) within PPI networks and bioactive compounds (Luteolinidin). It is usually considered that if the binding energy obtained by molecular docking is less than ⁃5 kcal/mol, it indicates that the target and the compounds have some binding activity, and the lower the binding energy, the more stable the binding between the two. Our docking analysis revealed that the binding energies of SRC, EGFR, and AKT1 with Luteolinidin were -4.9 kcal/mol, -8.6 kcal/mol, and -5.8 kcal/mol, respectively. The binding energy calculation from molecular docking inherently has a certain error range (typically ±1–2 kcal/mol). Therefore, -4.9 kcal/mol and-5.0 kcal/mol are statistically indistinguishable. Additionally, we analyzed the binding mode and found that LYS-195 and LYS-200 in the SRC protein are key binding sites. The ligand inserts into the protein in a specific conformation, utilizing the side chains of amino acid residues (especially lysine) to form non-covalent bonds such as hydrogen bonds and van der Waals forces, thereby stabilizing the complex structure. These results suggest that the interactions between Luteolinidin and these targets may play a crucial role in the treatment of GI using AAEM-V.

An inherent limitation of network pharmacology and molecular docking is the potential for off-target effects. The compounds we predict may exhibit stronger or weaker binding affinities with other proteins we have not analyzed, and their actual mechanisms of action may be more complex than the networks we have predicted. Therefore, the current docking results should primarily serve as a powerful hypothesis-generation tool, providing focused directions and theoretical basis for subsequent experimental validation (such as surface plasmon resonance SPR, enzyme activity inhibition assays, Western blotting, etc.), rather than being regarded as definitive evidence for mechanism validation.

### 3.8 Inhibition of cell proliferation and induction of apoptosis by active ingredients

**Figure 8:**
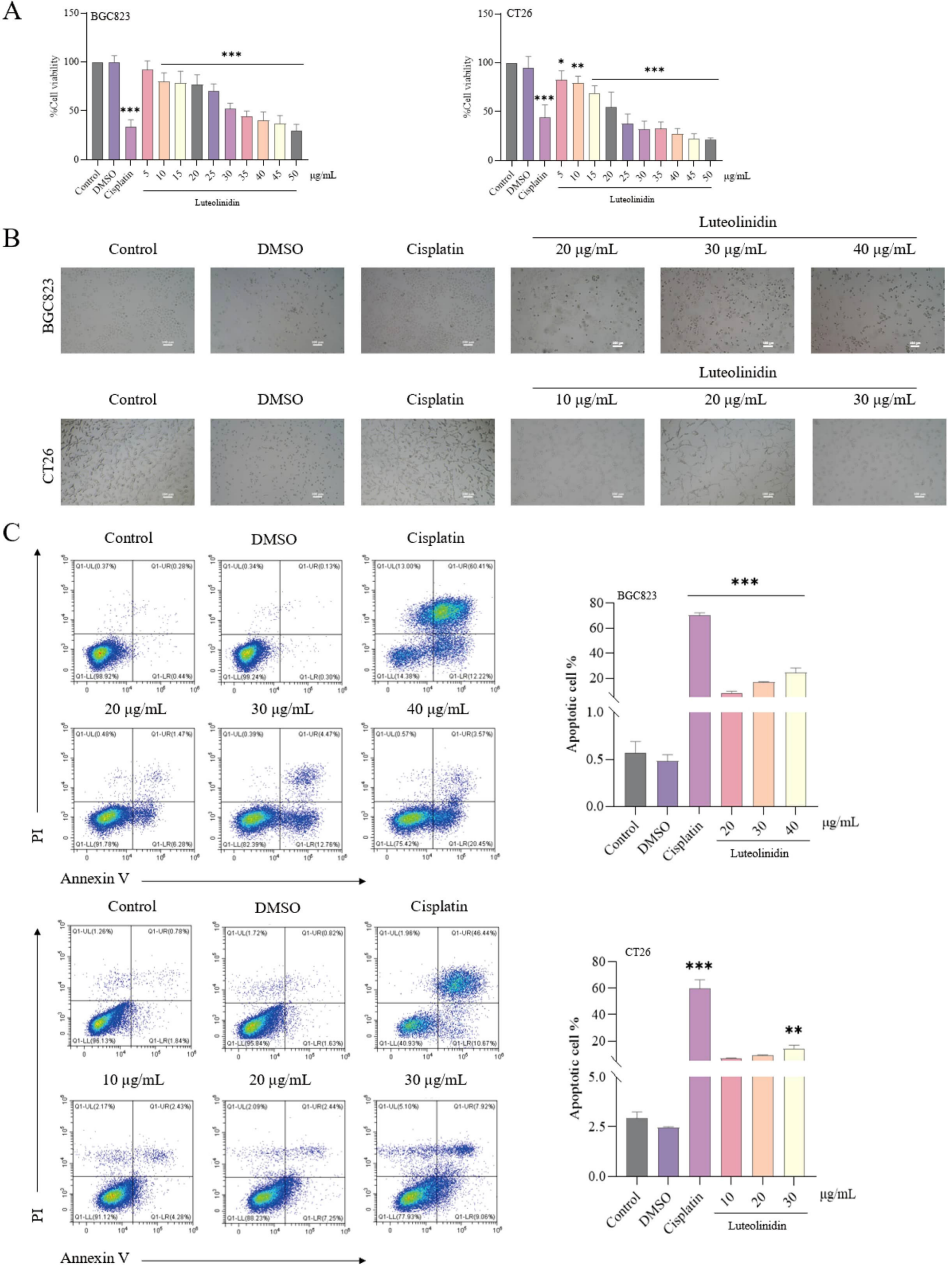
Luteolinidin can inhibit cell proliferation and induce apoptosis. **(A)** The viability of cells was detected by MTT assay. **(B)** The morphology of cells was observed by inverted microscopy. **(C)** After staining with Annexin V and PI, the cells were analyzed by flow cytometry. Data were ana-lyzed by one-way ANOVA, compared with control, *p<0.05, **p<0.01, ***p<0.001.

The antiproliferative effect of Luteolinidin was assessed by MTT method (**Figure 8A**) and the IC_50_ values of the drug were 33.2133 ± 3.438 μg/mL and 22.78 ± 2.5212 μg/mL for BGC823 and CT26 cells, respectively. Significant changes in the morphology of the cells and the de-crease in the density of the cells before and after the treatment of the drug were observed by inverted microscope and both of them showed dose dependence (**Figure 8B**). Flow cytometry analysis, coupled with Annexin V/PI dual staining, was employed to quantify apoptotic populations, revealing a notable increase in early and late apoptotic cells following Luteolinidin treatment (**Figure 8C**).

### 3.9 Induction of cell cycle arrest in S phase by active ingredients

**Figure 9:**
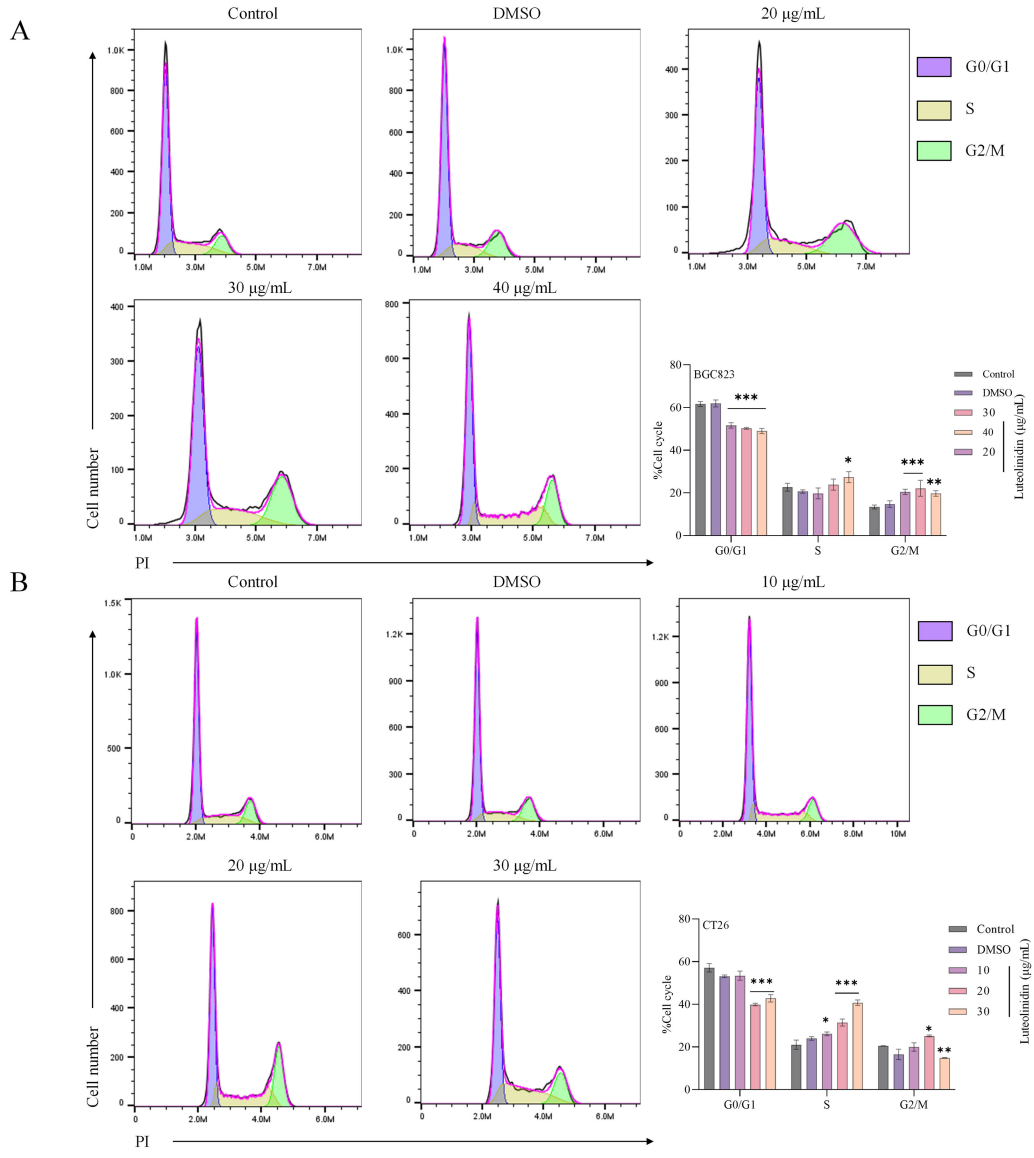
Luteolinidin induces cell cycle arrest in S phase. **(A)** BGC823 cells. **(B)** CT26 cells. Data were analyzed by one-way ANOVA, compared with control, *p<0.05, **p<0.01, ***p<0.001.

As shown in **Figure 9**, flow cytometric analysis revealed that Luteolinidin induced significant cell cycle arrest at the S phase, as evidenced by a notable increase in the proportion of cells in S phase (p<0.001) and a corresponding decrease in G1 and G2/M phases. This effect was dose-dependent, with maximal arrest observed at high concentration after 24 hours of treatment.

### 3.10 Induction of ROS accumulation by active ingredients

**Figure 10:**
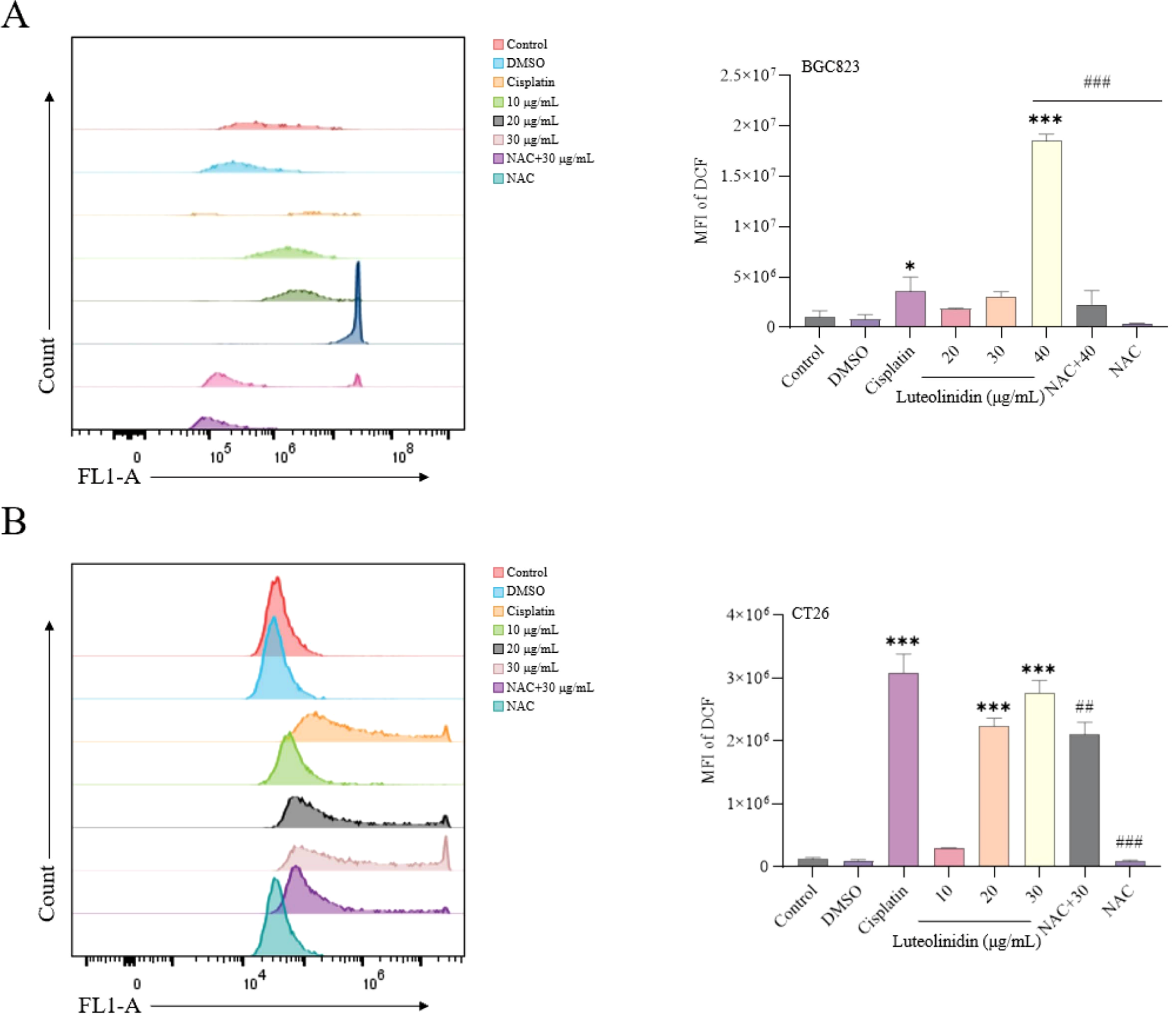
Induction of ROS accumulation by Luteolinidin. **(A)** BGC823 cells. **(B)** CT26 cells. Data were analyzed by one-way ANOVA, compared with control, *p<0.05, **p<0.01, ***p<0.001, compared with high concentrations, ##p<0.01, ###p<0.001.

Luteolinidin treatment significantly elevated intracellular reactive oxygen species (ROS) levels, as measured by flow cytometry using the fluorescent probe DCFH-DA (**Figure 10**). A dose-dependent increase in mean fluorescence intensity (MFI) was observed, with significant enhancement of fluorescence at high concentrations compared to untreated controls (p < 0.001). It was also found that drug treatment significantly reversed the effects of the ROS inhibitor NAC. It suggested that Luteolinidin can affect the intracellular oxidative stress response by promoting the elevation of cellular reactive oxygen species level, which leads to apoptosis.

### 3.11 Induction of a decrease in mitochondrial membrane potential by active ingredients

**Figure 11:**
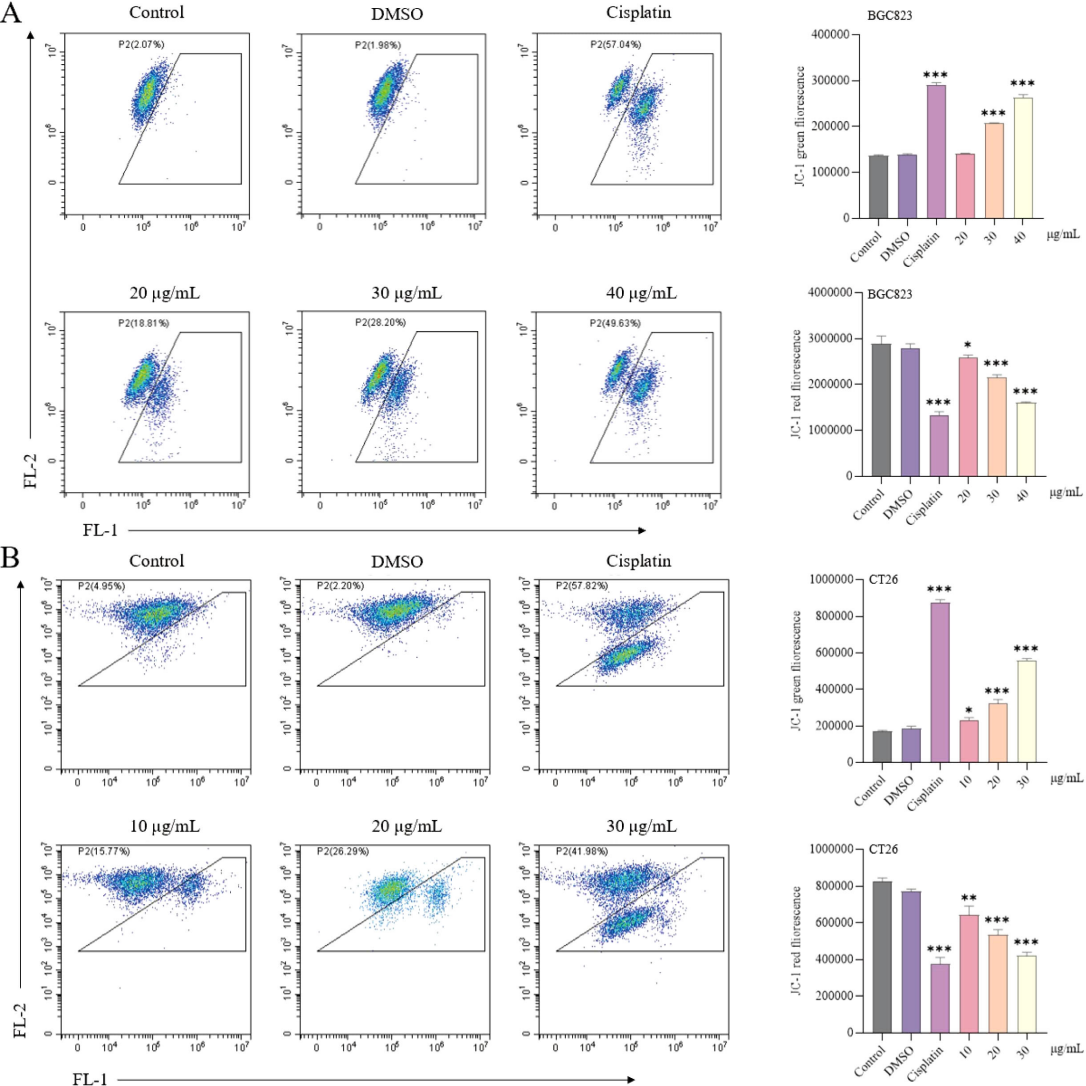
Induction of a decrease in mitochondrial membrane potential by Luteolinidin. **(A)** BGC823 cells. **(B)** CT26 cells. Data were analyzed by one-way ANOVA, compared with control, *p<0.05, **p<0.01, ***p<0.001.

Treatment with Luteolinidin significantly reduced mitochondrial membrane potential (ΔΨm) in BGC823 and CT26 cells, as determined by JC-1 staining and flow cytometry analysis. Red fluorescence (aggregated JC-1) was reduced and green fluorescence (monomeric JC-1) was elevated in the drug-treated group compared with the control group, indicating a decrease in mitochondrial transmembrane potential (**Figure 11**).

## 4 Discussion

A mounting body of evidence has demonstrated the efficacy of Chinese traditional medicine in the prevention of tumorigenesis and cancer progression (37, 38). Natural products have attracted significant attention in the field of oncology; indeed, it is estimated that almost 60% of clinically approved anticancer drugs are associated with natural products (39). Some studies have reported that Chinese herbs can exert anticancer effects through a variety of mechanisms, including inhibiting tumor cell proliferation, inducing apoptosis, modulating the immune system and reversing drug resistance (25). Hwang et al. (40) found that Oridonin increases the killing of lung cancer cells by activating NK immune cells to achieve anti-tumor effects. Huang et al. (41) showed that Betulinaldehyde causes apoptosis in lung cancer cells by regulating the autophagy pathway. *A. absinthium* is rich in total flavonoids, which can significantly inhibit the proliferation of cervical cancer cells and induce apoptosis (42). Meanwhile, *A. absinthium* ethanol extract (AAEE) has been identified as a promising novel therapeutic agent for cancer (42–44). In our study, we demonstrated that AAEM-V could lead to tumor cell death by promoting elevated levels of reactive oxygen species in gastrointestinal tumor cells, leading to a decrease in mitochondrial membrane potential and exhibited good inhibition of colorectal cancer development *in vivo*.

In addition, the antitumor activity of Luteolinidin, the active ingredient in AAEM-V, was evaluated. The results showed that Luteolinidin has good anti-tumor activity against gastrointestinal tumors. He et al. (45) proposed that *Sanghuangprous vaninii* extract inhibited the proliferation of cervical cancer cells through the endoplasmic reticulum stress-mitochondrial apoptosis pathway. Wei et al. (44) found that *A. absinthium* ethanol extract (AAEE) and its subfractions (AAEE-PE and AAEE-Ea) induced apoptosis and inhibited hepatocellular carcinoma cell growth via endoplasmic reticulum stress and mitochondria-dependent pathways. Apoptosis (also known as programmed cell death) has been demonstrated to exert a pivotal function in numerous disease-related biological processes (46, 47). It is regarded as a significant target for cancer therapy. During the initial phases of apoptosis, Annexin V exhibits a high degree of affinity for membrane phosphatidyl-serine (PS), which is translocated from the inner cell membrane to the cell surface (48). In contrast, in cells with plasma membrane damage, PI molecules can be observed interspersed within the DNA double helix (49). It can therefore be concluded that cells exhibiting strong staining with Annexin V are indicative of early apoptosis, whereas PI-stained cells are suggestive of late apoptosis or necrosis. The present study demonstrates that Luteolinidin can induce apoptosis. Reactive oxygen species (ROS) are a class of molecules that have been shown to play a critical role in determining cell fate (50). The relationship between ROS and tumor biology is intricate (51). ROS plays an important role in cellular function and survival signaling, and the accumulation of intracellular ROS could finally activate apoptosis signaling pathways (52). Recent studies have demonstrated the importance of ROS in therapeutic approaches to effectively kill various tumor cells (53). Mavrikou et al. (54) found that *Viscum album* L. extract caused oxidative changes and induced apoptosis by activating caspase-3 and promoting elevated intracellular ROS levels. Pérez-Sánchez et al. (55) found that Rosmarinus officinalis extract led to a dramatic increase in intracellular ROS in colorectal cancer cells and induced apoptosis. Consistent with the results of this study, Luteolinidin induced the release of ROS. The cell cycle is the central biological process of cell growth, proliferation and differentiation, and is regulated by a series of stringent checkpoints (G1/S, G2/M) to ensure the accuracy of DNA replication and genomic stability. Tumorigenesis is often closely related to cell cycle dysregulation. Hung et al. (56) found that Bavachinin induced G2/M cell cycle arrest and apoptosis via the ATM/ATR signaling pathway in Human Small Cell Lung Cancer. As we expected, Luteolinidin induced apoptosis in BGC823 and CT26 cells by cycle blocking in S phase.

This study confirmed that *A. absinthium* L. extract (AAEM-V) and its main active component, Luteolinidin, can inhibit the growth of colorectal cancer cells by inducing ROS production, mitochondrial dysfunction, and cell cycle arrest. Notably, one of the key values of natural products lies in their ability to reverse chemotherapy resistance in tumor cells and produce synergistic effects with other drugs, which is of great significance for improving clinical treatment outcomes. Recent studies have provided new mechanistic insights and strategic options for natural products to reverse chemotherapy drug resistance. Song et al. (57) found that the combination of Saikosaponin-a and gemcitabine exhibits a significant synergistic enhancement effect in the treatment of intrahepatic cholangiocarcinoma, highlighting the immense potential of natural products as antibiotic “potentiators.”

Although this study revealed the antitumor potential of AAEM-V, we must acknowledge that it has certain limitations, mainly due to tumor heterogeneity. The functional validation in this study was conducted exclusively in two cell lines: BGC823 (human gastric cancer) and CT26 (mouse colon cancer). *In vivo* experiments were performed using only the CT26 syngeneic tumor model. This limited model selection implies that our findings may not be directly applicable to other cancer types (e.g., breast cancer, lung cancer) or even different molecular subtypes within the same cancer (e.g., EBV-positive, MSI-positive, GS-positive subtypes of gastric cancer). Different cancer subtypes typically exhibit distinct driver gene mutations and signaling pathway activation states, which may result in potential variability in their therapeutic response to AAEM-V. Therefore, our conclusions primarily apply to specific models of colorectal and gastric cancer.

## 5 Conclusions

In this study, we found that AAEM-V was effective in inhibiting the development of gastrointestinal tumors *in vitro* and *in vivo*, and at the same time, its toxicity and side effects were greatly reduced compared with cisplatin. Luteolinidin exerts potent antitumor effects against gastrointestinal cancer cells by suppressing proliferation, elevating intracellular reactive oxygen species (ROS) levels, reducing mitochondrial membrane potential (ΔΨm), inducing S-phase cell cycle arrest, and ultimately triggering programmed cell death. These findings provide a scientific basis for the traditional use of *A. absinthium* in cancer therapy and high-light its potential as a source of novel chemotherapeutic agents.

## Data Availability

The original contributions presented in the study are included in the article/**Supplementary Material**. Further inquiries can be directed to the corresponding author/s.
