# Supplementary material for: Artemisia absinthium L. ethanol extract inhibits the growth of gastrointestinal cancer cells by inducing apoptosis and mitochondria-dependent pathway
Source: Front Oncol. 2025 Oct 27;15:1644498. doi: 10.3389/fonc.2025.1644498 (PMC12597748; doi:10.3389/fonc.2025.1644498)
Supplement: Supplementary file 1 [file DataSheet1.docx]

**A Total Ion Chromatogram from 85% sample：**
